# Role of Macrophages in the Pathogenesis of Genotype VII Newcastle Disease Virus in Chickens

**DOI:** 10.3390/ani13132239

**Published:** 2023-07-07

**Authors:** Jie Ni, Jing Deng, Qing Chen, Tianxing Liao, Jiao Hu, Yu Chen, Shunlin Hu, Zenglei Hu, Xiufan Liu

**Affiliations:** 1 Key Laboratory of Animal Infectious Diseases, School of Veterinary Medicine, Yangzhou University, Yangzhou 225009, China; jenniezs@163.com (J.N.); slhu@yzu.edu.cn (S.H.); 2 Jiangsu Co-Innovation Center for Prevention and Control of Important Animal Infectious Diseases and Zoonosis, Yangzhou University, Yangzhou 225009, China; 3 Jiangsu Key Laboratory of Zoonosis, Yangzhou University, Yangzhou 225009, China; 4 Joint International Research Laboratory of Agriculture and Agri-Product Safety, The Ministry of Education of China, Yangzhou University, Yangzhou 225009, China

**Keywords:** Newcastle disease virus, macrophages, depletion, replication, tissue damage, pathogenesis

## Abstract

**Simple Summary:**

Genotype VII Newcastle disease virus (NDV) is renowned for its high tropism to the immune organs of chickens, and macrophages are a critical component in the antiviral innate immune system. The objective of this study is to investigate the role of macrophages in the pathogenesis of genotype VII NDV in chickens. Genotype VII NDV has significantly higher infectivity in macrophages compared to genotype IV NDV. Macrophage depletion resulted in no changes in virulence and virus replication but a significant deterioration of tissue damage in the spleen. Therefore, macrophages play an important role in alleviating tissue lesions caused by genotype VII NDV infection.

**Abstract:**

Long-term evolution of Newcastle disease virus (NDV) results in substantial alteration in viral pathogenesis. NDVs of genotype VII, a late genotype, show marked tropism to lymphoid tissues, especially to macrophages in chickens. However, the role of macrophages in the pathogenesis of genotype VII NDV is still unclear. Herein, NDV infectivity in macrophages and the role of macrophages in the pathogenesis of genotype VII NDV in chickens were investigated. We reported that NDV strains of genotype VII (JS5/05) and IV (Herts/33) can replicate in the adherent (predominantly macrophages) and non-adherent cells (predominantly lymphocytes) derived from chicken peripheral blood mononuclear cells (PBMCs), and significantly higher virus gene copy was detected in the adherent cells. In addition, JS5/05 had significantly higher infectivity in PBMC-derived adherent cells than Herts/33, correlating with its enhanced tropism to macrophages in the spleen of chickens. Interestingly, the depletion of 68% of macrophages exerted no significant impact on clinical signs, mortality and the systematic replication of JS5/05 in chickens, which may be associated with the contribution of non-depleted macrophages and other virus-supportive cells to virus replication. Macrophage depletion resulted in a marked exacerbation of tissue damage and apoptosis in the spleen caused by JS5/05. These findings indicated that macrophages play a critical role in alleviating tissue damage caused by genotype VII NDV in chickens. Our results unveiled new roles of macrophages in NDV pathogenesis in chickens.

## 1. Introduction

Newcastle disease (ND) is an acute and highly contagious infectious disease caused by velogenic Newcastle disease virus (NDV). ND causes high mortality in a variety of poultry species and is a serious threat to the development of the poultry industry. NDV is a member of the genus *Orthoavulavirus* in the family *Paramyxoviridae* [1]. Velogenic NDV strains induce systematic infection and severe tissue lesions in the respiratory, gastrointestinal, neurological, reproductive and immune systems of poultry [2,3], suggesting viral tropism to a wide range of cell types.

NDV can replicate in various cell types such as epithelial cells, macrophages, dendritic cells and glial cells (neurons, astrocytes, oligodendrocytes and microglia) [4,5,6,7,8]. Specifically, in NDV-infected chickens, extensive distribution of the viral antigens can be detected in macrophage-like cells in lymphoid tissues, indicating the tropism of NDV to macrophages [9,10]. In addition, independent in vitro studies explored the mechanism of NDV infectivity in macrophages from various perspectives. A recent study showed that NDV entry into chicken macrophages is associated with a pH-dependent, dynamin- and caveola-mediated endocytosis pathway, and Rab5 is required for this process [8]. Velogenic NDV induces an M1-/M2-like mixed polarized activation of chicken macrophages through the inhibition of toll-like receptor 7, resulting in enhanced replication compared to lentogenic viruses [11]. Additionally, the fusion and hemagglutinin-neuraminidase proteins are determinants for the macrophage host range of NDV [12]. These studies suggested that macrophages, the main target cells for NDV infection, may play an important role in viral pathogenesis.

NDV has undergone substantial evolution since its emergence in 1926, resulting in prominent alternation of the pathogenesis. Genotype VII NDV, a late genotype emerging in the 1990s [13,14,15], shows high tropism to lymphoid tissues of chickens compared to strains of early genotypes, such as genotype IV. The induction of an aberrant innate immune response contributes to the pathology of the genotype VII virus [16,17,18]. Macrophages, as a key component of the innate immune system, play a crucial antiviral role through clearing apoptotic cells or virus-infected cells and producing cytokines. Additionally, extensive distribution of the viral antigen in macrophage-like cells and remarkable necrosis of these cells were detected in the spleen of chickens infected with genotype VII NDV [9]. Therefore, the critical role of macrophages in the innate immune response and high tropism of genotype VII NDV to this cell type prompted us to hypothesize that macrophages may be engaged in determining the pathogenesis of genotype VII NDV in chickens.

In this study, we reported that genotype VII NDV had higher infectivity in macrophages in vitro and in vivo compared to the genotype IV virus. Macrophage depletion had no influence on the disease outcomes or replication of genotype VII NDV in chickens but exacerbated virus-induced tissue damage and apoptosis in the spleen. This indicates a critical role of macrophages in protection against pathology caused by NDV in chickens. Our results identified new roles of macrophages in the pathogenesis of genotype VII NDV in chickens.

## 2. Materials and Methods

### 2.1. Cells, Viruses and Plasmid

Chicken peripheral blood mononuclear cells (PBMCs) were isolated from 2-month-old specific pathogen-free (SPF) white leghorn chickens through density gradient centrifugation using a chicken PBMC isolation kit (TBD Science, Tianjin, China). PBMCs were grown in RPMI 1640 medium supplemented with 10% fetal bovine serum (FBS) (ThermoFisher Scientific, Waltham, MA, USA) at 37 °C, 5% CO_2_. Chicken embryo fibroblasts (CEFs) were prepared using 9-day-old SPF chicken embryos and cultured in M199 medium supplemented with 4% FBS. Genotype VII NDV JS5/05 and genotype IV NDV Herts/33 were reported previously [16] and were propagated in 9-day-old SPF embryonated chicken eggs (ECEs). Virus titers were measured as 50% tissue culture infectious dose (TCID_50_) in CEFs and as 50% embryo infectious dose (EID_50_) in ECEs. The plasmid containing the full-length genome of JS5/05 was generated previously [19], and was used as a standard plasmid in absolute quantitative real-time PCR (qRT-PCR) for virus titration.

### 2.2. Isolation and Identification of Macrophages from PBMCs

Blood was collected from three 2-month-old SPF white leghorn chickens, pooled and used for PBMC isolation. Macrophages were isolated from PBMCs as previously reported [20]. PBMCs were seeded in a 6-well plate, and the non-adherent cells were removed by washing with PBS at 2 and 24 h after seeding. To determine the ratio of macrophages, the adherent cells were trypsinized to prepare single cells, and 1 × 10^7^ single cells were labeled with FITC-conjugated mouse anti-chicken KUL01 antibody (1:100) (SouthernBiotech, Birmingham, AL, USA) for 45 min at 4 °C in FACS buffer (PBS containing 2% FBS). Then, the cells were washed and resuspended in PBS, and 1 × 10^4^ cells were used for flow cytometry analysis.

### 2.3. In Vitro Infectivity of NDV in the Adherent and Non-Adherent Cells Derived from PBMC

To assess NDV infectivity in different cell fractions derived from PBMCs, 1 × 10^7^ PBMCs were seeded in 6-well culture plates and then inoculated with JS5/05 or Herts/33 at a multiplicity of infection (MOI) of 0.01. At 8, 16 and 24 h post inoculation (p.i.), the adherent (predominantly macrophages) and non-adherent cells (predominantly lymphocytes) were separated, and total RNA was extracted from the cells and transcribed into cDNA using a PrimeScript RT Reagent Kit with gDNA Eraser (Takara, Shiga, Japan). Virus replication/transcription in the cells was determined by measuring the copy of the *fusion* (*F*) gene using qRT-PCR. The *F* gene was amplified using a pair of primers (sense: 5′-GGTCAATCATAGTCAAGTTGCTCC-3′; anti-sense: 5′-AACCCCAAGAGCTACACTGCC-3′) and a TaqMan probe (5′-FAM-AAGCGTYTYTGTCTCCTTCCTCC-BHQ-3′). The primers and the probe were designed according to the sequences of JS5/05 (accession no. JN631747.1) and Herts/33 (accession no. AY741404.1). In brief, the standard plasmid was 10-fold serially diluted, and the samples containing 10^2^ to 10^9^ copies were used to plot the standard curve. The qRT-PCR reaction system was composed of 10 μL of 2 × AceQ Universal U + Probe Master Mix (Vazyme, Nanjing, China), 0.7 μL of the sense and anti-sense primers (final concentration 0.35 μM), 0.7 μL of the probe, 2 μL of the cDNA and 5.8 μL of deionized water. PCR reactions were performed in triplicate using a LightCycler^®^ 480 (Roche, Basel, Switzerland) with the following cycle profile: 1 cycle at 95 °C for 10 min followed by 40 cycles at 95 °C for 15 s and 60 °C for 35 s. The *F* gene copy in the NDV-inoculated cells was calculated based on the standard curve. The experiments were performed in triplicate.

### 2.4. In Vivo Infectivity of NDV in Macrophages

To determine NDV infectivity in macrophages in vivo, chickens were infected with NDV, and virus replication in macrophages in the spleen was measured using immunofluorescence assays (IFA). Briefly, nine 5-week-old SPF chickens were randomly divided into three groups, with three chickens per group. Three SPF chickens were inoculated with 10^5^ EID_50_ of JS5/05 or Herts/33 via the intranasal and intraocular routes. Another three chickens were inoculated with PBS as the mock control. On day 3 p.i., the chickens were euthanized, and the spleens were collected for the preparation of formalin-fixed paraffin-embedded sections. The nucleoprotein (NP) protein of NDV was detected using mouse anti-NP monoclonal antibody (a gift from Dr. Chan Ding, Shanghai Veterinary Research Institute, China) and the macrophage marker KUL01 was probed using mouse anti-chicken KUL01 antibody (SouthernBiotech). The cell nucleus was stained with 2-(4-Amidinophenyl)-6-indolecarbamidine dihydrochloride (DAPI) (Beyotime, Nantong, China). The entire spleen section was scanned and the images were processed and analyzed using the CaseViewer software (version 2.3).

### 2.5. Macrophage Depletion in Chickens

Clodronate liposomes (CL lipo) (Yeasen, Shanghai, China) were used for macrophage depletion in chickens as reported previously [21]. Six 5-week-old SPF chickens were randomly divided into two groups, with three chickens in each group. Three 5-week-old SPF chickens were intravenously (i.v.) treated with 250 μL of CL lipo or the control reagent, PBS liposomes (PBS lipo), respectively. The efficiency of macrophage depletion was verified using IFA in the spleen on day 5 post-treatment (pt). The spleens were fixed with 10% buffered formalin and prepared into tissue sections. A mouse anti-chicken KUL01 monoclonal antibody (1:50) was added to the sections as the primary antibody and incubated at 4 °C overnight. After washing with PBS 3 times, the sections were then incubated with Alexa Fluor 647-conjugated goat anti-mouse antibody as the secondary antibody (1:200) (ThermoFisher Scientific) at 37 °C for 1 h. After washing with PBS, the cell nucleus was stained with DAPI. The images were captured using a fluorescence microscope (Leica).

### 2.6. Chicken Infection Study

Twenty-seven 5-week-old SPF chickens were randomly divided into three groups, nine chickens per group. Two groups of chickens were i.v. treated with 250 μL of CL lipo or PBS lipo, and chickens in the third group were i.v. inoculated with 250 μL of PBS as the mock control. Two days later, the CL lipo- and PBS lipo-treated chickens were inoculated with 10^5^ EID_50_ of JS5/05 via the intranasal and intraocular routes. The mock chickens were inoculated with PBS via the same route. On days 1 and 3 p.i., three chickens per group were euthanized, and the liver, spleen, lung, thymus and intestine were collected. The remaining three chickens of each group were observed daily for clinical signs and mortality. All the tissues were used for virus load measurement. The tissue samples were homogenized in PBS supplemented with antibiotics (1 mg tissue in 0.3 mL PBS), and the homogenates were centrifugated to collect the supernatants. The supernatants were 10-fold serially diluted using M199 medium supplemented with 1% FBS and were inoculated into CEFs. At 72 h post inoculation, the culture supernatants of the cells were harvested and subjected to hemagglutination assays to determine virus infection. Virus titers were calculated as TCID_50_ per gram using the Reed and Muench method [22].

### 2.7. Histopathological Analysis

The spleens were selected for histopathological assessment because JS5/05 causes remarkable lesions in this tissue. The spleens collected on days 1 and 3 p.i. were fixed with 10% buffered formalin, prepared into tissue sections and stained with hematoxylin–eosin (HE). The histopathological changes were scored as reported previously [16]: −, normal; +, mild to moderate lymphocyte proliferation; ++, moderate to marked lymphocyte depletion and necrosis; +++, marked lymphocyte depletion, necrosis and tissue hyperplasia; ++++, extensive and severe lymphocyte depletion and necrosis.

### 2.8. TUNEL Staining

To evaluate whether macrophage depletion impacts apoptosis induced by NDV, the spleen tissues were examined using a terminal deoxnuceotidyl transferase-mediated dUTP biotin nick end labeling (TUNEL) kit according to the manufacturer’s instructions. Briefly, 50 μL of equilibration buffer was added to the tissue section and incubated at room temperature for 10 min. After removing the equilibration buffer, 56 μL of labeling solution containing recombinant TdT enzyme, FITC-12-dUTP labeling mix and equilibration buffer was added to the tissues and incubated at 37 °C for 1 h. After washing with PBS, the tissue sections were incubated with DAPI for nucleus staining. The images were captured using a fluorescence microscope (Leica).

## 3. Data analysis

The pathological and immunofluorescence images were processed and analyzed using CaseViewer and ImageJ software (1.52V), and the flow cytometry data were analyzed using FlowJo software (V10). Statistical analysis of the data was performed using GraphPad Prism. The data are presented as the mean values ± standard deviation (SD) of three independent experiments. Statistical differences were analyzed using the *t*-test or one-way ANOVA. *p* < 0.05 was considered a significant difference.

## 4. Results

### 4.1. NDV Has Higher Infectivity in Macrophages than Lymphocytes

**Figure 1:**
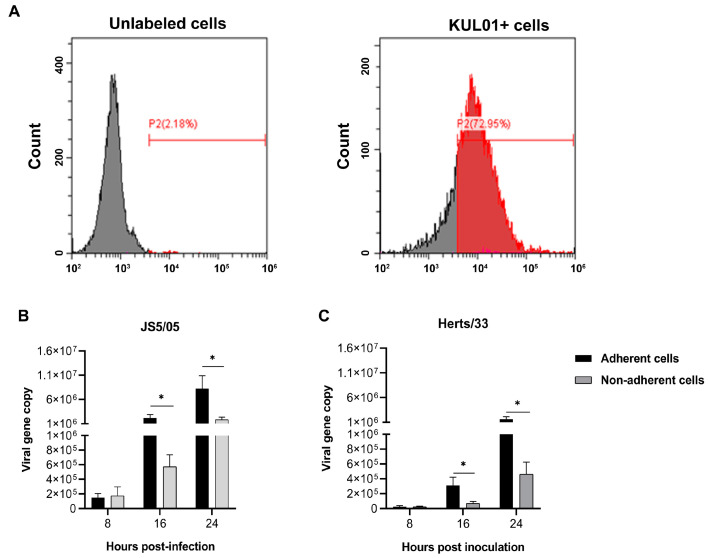
Ratio of macrophages in chicken PBMCs and NDV infectivity in different fractions of PBMCs. (**A**) Percentage of KUL01-positive cells in chicken PBMCs. The adherent cells were isolated from chicken PBMCs and the percentage of KUL01-positive cells in the adherent cells was determined using flow cytometry. Left panel: unlabeled cells; right panel, KUL01-positive cells. (**B**,**C**) NDV replication/transcription in the adherent and non-adherent cells derived from PBMCs. (**B**) JS5/05; (**C**) Herts/33. PBMCs were inoculated with NDV at a 0.01 multiplicity of infection, and at 8, 16 and 24 post-inoculation, the adherent and non-adherent cells were separated. The *fusion* gene copy was measured in these two cell fractions using quantitative real-time PCR. The data are presented as the mean values ± standard error of three independent experiments. Asterisk (*) stands for a significant difference (*p* < 0.05).

The infectivity of the genotype VII and IV NDV strains in macrophages was assessed in vitro. As previously reported, the majority of the adherent and non-adherent cells derived from PBMCs or splenocytes are macrophages and lymphocytes, respectively [12,20,21]. Herein, macrophages were isolated from chicken PBMCs, and NDV replication/transcription in these cells was determined using qRT-PCR. A high percentage of the adherent cells (72.9%) derived from PBMCs were KUL01-positive (Figure 1A), indicating that the adherent cells were predominantly macrophages. Viral gene copies of JS5/05 and Herts/33 in the adherent and non-adherent cells steadily increased from 8 to 24 h p.i., suggesting that these two cell fractions were supportive for NDV replication/transcription (Figure 1B,C). Overall, JS5/05 had significantly higher virus gene copy in the adherent and non-adherent cells compared to Herts/33 (Figure 1B,C). In addition, at 16 and 24 h p.i., significantly higher viral copy of both NDV strains were detected in the adherent cells compared to those in the non-adherent cells (Figure 1B,C). These data demonstrated that the virus replication/transcription of the JS5/05 and Herts/33 strains was significantly higher in adherent cells compared to non-adherent cells, and JS5/05 had higher infectivity in both cell fractions compared to Herts/33.

### 4.2. Genotype VII NDV Presents an Enhanced Tropism to Macrophages in Chickens

**Figure 2:**
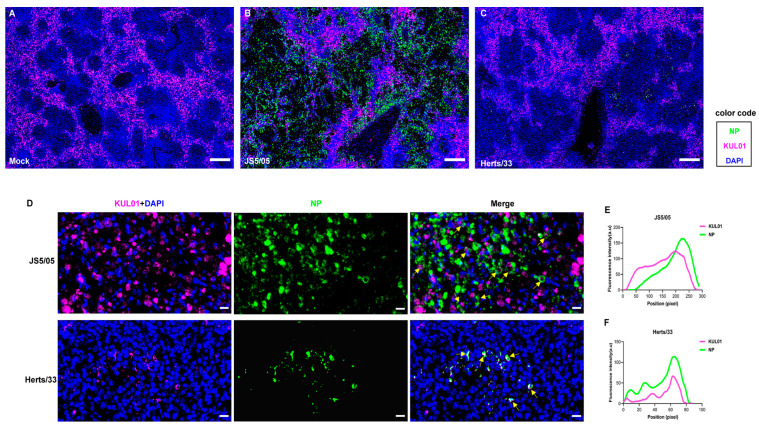
NDV tropism to macrophages in the spleen. (**A**–**C**) Replication of NDV in the spleen of chickens. (**A**) The non-infected mock chickens; (**B**) the JS5/05-infected chickens; (**C**) the Herts/33-infected chickens. Chickens were infected with JS5/05 or Herts/33, and on day 3 post-infection, the spleens were harvested for preparation of tissue sections. Virus replication in the spleen was assessed using immunofluorescence assays. The NP protein of NDV (green) and the marker of chicken macrophages KUL01 (magenta) were detected, and the cell nucleus was stained with DAPI (blue). The entire spleen sections were scanned, and representative fields of the spleen sections of three chickens are shown. Scale bar, 200 µm. (**D**) Virus tropism to macrophages. The NP antigen and KUL01 signals in the representative regions in the spleen from the NDV-infected chickens are shown. Co-localization of the NP and KUL01 was observed. Co-localization of the NP and KUL01 is indicated by arrows. Scale bar, 10 µm. (**E**) Co-localization profiles of the NP and KUL01 in JS5/05-infected spleen; (**F**) co-localization profiles of the NP and KUL01 in Herts/33-infected spleen.

To further verify NDV infectivity in macrophages in vivo, chickens were infected with NDV, and viral tropism to macrophages in the spleen was determined using IFA. No NP antigen was detected in the spleen of the mock-infected chickens (Figure 2A). An extensive distribution of the NP antigen in the spleen was observed in the JS5/05-infected chickens (Figure 2B), while the NP-positive area was markedly limited in the Herts/33-infected spleens (Figure 2C). In addition, in the virus-infected chicken spleens, the majority of the NP-positive cells were positive for the macrophage marker KUL01 (Figure 2D). The co-localization of the NP antigen and KUL01 marker verified virus tropism to macrophages in vivo (Figure 2E,F). These results showed that NDV exhibited tropism to macrophages in the spleen, and the genotype VII NDV strain JS5/05 presented enhanced infectivity in macrophages compared to the genotype IV NDV strain Herts/33.

### 4.3. Clodronate Liposome Treatment Causes Efficient Macrophage Depletion

**Figure 3:**
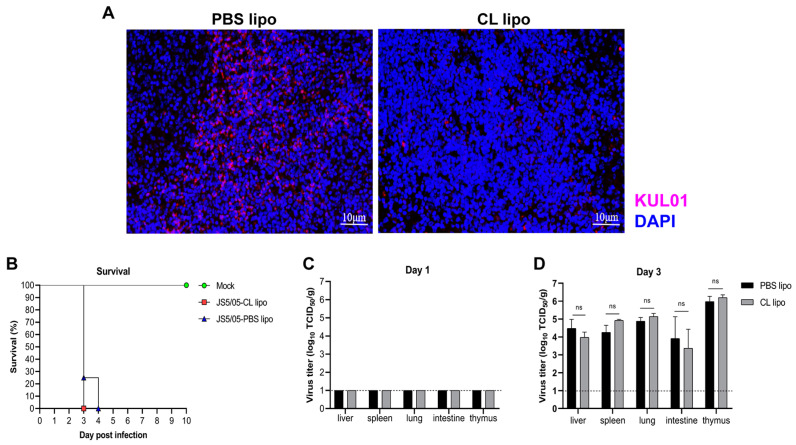
Impact of macrophage depletion on NDV pathogenicity and replication in chickens. (**A**) Efficiency of macrophage depletion in the spleen. Chickens were treated with clodronate liposomes (CL lipo) or PBS liposomes (PBS lipo), and on day 5 post-treatment, the percentage of macrophages in the spleen was determined to determine the efficiency of macrophage depletion. Red, KUL01; blue, cell nucleus. Scale bar, 10 µm. (**B**) Survival of the chickens treated with CL lipo or PBS lipo after NDV infection. On day 2 after the treatment with CL lipo or PBS lipo, chickens were inoculated with JS5/05 and animal survival was determined. (**C**,**D**) Virus load in the CL lipo- and PBS lipo-treated chickens. The liver, spleen, lung, intestine and thymus were collected on days 1 (**C**) and 3 (**D**) post-infection for measurement of viral load. The dotted lines indicate the detection limit of the virus titration assay (1.0 log_10_ TCID_50_/g). The data of virus load are presented as the mean values ± standard error of three independent experiments. ns, non-significant differences.

To investigate the contribution of macrophages to NDV pathogenicity, macrophages were depleted in chickens with treatment with CL lipo, a widely-used reagent for in vivo macrophage clearance. Chickens were i.v. injected with 250 μL of CL lipo, and on day 3 p.t., the number of KUL01-positive cells in the spleen of chickens treated with CL lipo decreased significantly compared to the PBS lipo-treated chickens (Figure 3A), resulting in a depletion efficiency of 68%. These data showed that clodronate liposome treatment resulted in efficient macrophage clearance in chickens.

### 4.4. Macrophage Depletion Does Not Alter Disease Outcomes and NDV Replication in Chickens

Subsequently, chickens were treated with CL lipo, and inoculated with JS5/05 two days later. The CL lipo- and PBS lipo-treated chickens displayed similar clinical signs after NDV infection such as severe depression, apparent respiratory signs and diarrhea, and rapidly died from virus infection within 4 days p.i. (Figure 3B). In addition, no infectious virus was recovered from the spleen, liver, lung, thymus or intestine of the CL lipo- and PBS lipo-treated chickens on day 1 p.i. (Figure 3C), and on day 3 p.i., virus replication was detected in all the tested tissues of the treated chickens (Figure 3D). However, there were no significant differences in virus load in the tested tissues between the chickens treated with CL lipo and PBS lipo (Figure 3D). Together, these results suggested that macrophage depletion did not affect the disease outcomes or systematic replication of NDV in chickens.

### 4.5. Macrophage Depletion Exacerbates Tissue Damage Caused by NDV

**Figure 4:**
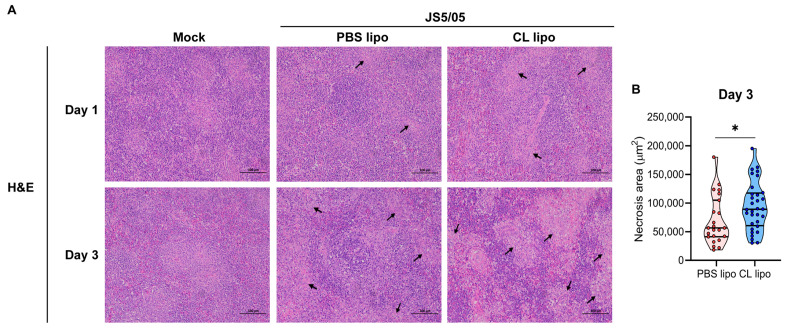
Histopathological changes caused by NDV in chicken spleens after macrophage depletion. (**A**) Pathological lesions of the spleens. The spleens of the NDV-infected chickens were collected and prepared into tissue sections, which were subjected to H&E staining. Upper panel, day 1 post-infection; lower panel, day 3 post-infection. Arrows indicate necrotic foci in the spleen. Scale bar, 100 µm. (**B**) Quantitation of the area of necrotic foci in the spleens. The area of necrosis in the spleens collected on day 3 post-infection was calculated using ImageJ software (1.52V). Asterisk (*) stands for a significant difference (*p* < 0.05).

As a key antiviral component, macrophages may be involved in the induction of the innate immune response and pathological lesions caused by NDV. To assess the role of macrophages in NDV pathogenicity, under the condition of macrophage depletion, tissue damage in the spleen caused by JS5/05 was assessed because this strain induces remarkable lesions in this organ. On day 1 p.i., no obvious pathological changes were seen in the non-infected chickens, and JS5/05 induced mild hemorrhage and lymphocyte necrosis in the chickens treated with CL lipo and PBS lipo (Figure 4A). On day 3 p.i., no tissue lesions were detected in the spleen of the non-infected chickens, whereas marked lymphocyte depletion and severe necrosis were observed in the JS5/05-infected chickens after PBS lipo treatment (Figure 4A). Of note, upon CL lipo treatment, JS5/05 infection caused more remarkable pathological changes in the spleen, characterized by enhanced lymphocyte depletion and large and extensive necrotic foci and hemorrhage, compared to that of the PBS lipo-treated chickens (Figure 4A,B) (Table 1). These findings showed that macrophage depletion deteriorated tissue lesion in the spleen caused by JS5/05, indicating a role of macrophages in alleviating tissue damage.

### 4.6. Macrophage Depletion Enhances Apoptosis Caused by NDV

**Figure 5:**
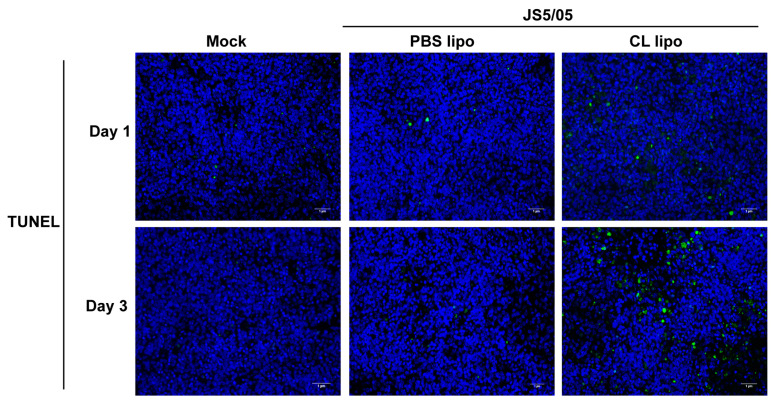
Apoptosis in the spleens of NDV-infected chickens after macrophage depletion. The spleen sections were used for TUNEL staining to detect apoptotic cells. Apoptotic cells and cell nuclei are shown in green and blue fluorescence, respectively. **Upper panel**, day 1 post-infection; **lower panel**, day 3 post-infection. Scale bar, 1 µm.

NDV infection induces apoptosis, and macrophages engulf apoptotic cells. To evaluate the impact of macrophage depletion on virus-induced apoptosis, apoptotic cells were detected using TUNEL staining in the spleen. On day 1 p.i., only a few apoptotic cells were detected in the spleen of the non-infected chickens and virus-infected chickens after PBS lipo treatment, and JS5/05 infection resulted in more apoptotic cells in the spleen of the CL lipo-treated chickens (Figure 5). On day 3 p.i., the number of apoptotic cells in the spleen of the chickens treated with CL lipo after JS5/05 infection was significantly increased compared with the PBS lipo-treated and non-infected chickens (Figure 5). Therefore, NDV-induced apoptosis in the spleen was exacerbated after macrophage depletion.

## 5. Discussion

Genotype VII NDV is characterized by its marked tropism to chicken lymphoid tissues, especially to macrophages. It seems to be plausible that macrophages, an important immune cell type, may play a critical role in regulating virus pathogenesis. Herein, we reported that NDV had higher tropism to the PBMC-derived adherent cells compared to non-adherent cells, and in particular, the genotype VII virus showed enhanced infectivity compared to the genotype IV virus in these cells. When macrophages were depleted by CL lipo treatment, no significant changes in the disease manifestation and virus replication of genotype VII NDV were detected, while virus-induced tissue damage was exacerbated. These results indicated that macrophages protect against pathology caused by NDV infection in chickens.

Macrophages, as an essential component in the innate immune system, are major target cells for NDV infection. Some studies in chickens showed that the viral antigen or nucleic acid of NDV was detected in multiple tissues, but most predominantly in macrophages associated with lymphoid tissues [9,10]. However, in these studies, the virus-infected cell type was assessed mainly based on histological observation. In the present study, a specific marker for chicken macrophages, KUL01, was used to identify the cell type of NDV infection. The genotype VII and IV strains could replicate in KUL01-positive cells in the spleen, confirming the tropism of NDV to macrophages. Of note, the NP antigen was predominantly found in macrophages in the ellipsoids in the white pulp but not in macrophages in the red pulp. This finding correlates with the previous results that NDV mainly replicates in the white pulp in the spleen [9,23,24]. The mechanism for the differential tropism of NDV to macrophages in the white and red pulps is still unknown. Moreover, several in vitro infection studies revealed the infectivity of NDV in primary macrophages or the macrophage cell line [11,12]. In these studies, velogenic strains were compared to lentogenic or mesogenic strains, suggesting that NDV tropism to macrophages correlates with the virulence. Here, we showed that two velogenic strains belonging to different genotypes had distinct infectivity to macrophages, with an enhanced tropism observed for the genotype VII virus. These results indicated that viral tropism to macrophages may change as a result of NDV evolution.

Based on the high tropism of genotype VII NDV to macrophages, the contribution of macrophages to virus replication in chickens was examined through cell depletion. CL lipo was used herein to deplete macrophages in chickens because this reagent is efficient for depleting macrophages in animals. The results showed that 68% of the KUL01-positive cells in the spleen were depleted, and this efficiency for macrophage clearance was comparable to those in previous reports [21,25,26]. Efficient depletion of macrophages is fundamental to examine their roles in NDV pathogenicity. Macrophage depletion resulted in no significant differences in virus load in chicken tissues, indicating that macrophage depletion did not affect the systematic infection of NDV. Similarly, the depletion of macrophages from the lungs via intranasal inoculation of CL lipo did not affect the replication of Middle East respiratory syndrome coronavirus (MERS-CoV) in mice [27]. In contrast, the replication of Marek’s disease virus (MDV) and H3N2 influenza virus in the host was significantly increased after CL lipo treatment [28,29]. The findings obtained in our study seem to be conflicting with the high infectivity of NDV in macrophages. There are three possible explanations for these results. First, after CL lipo treatment, there are still 32% of macrophages remaining in the spleen. This fraction of macrophages is still supportive for NDV infection and may contribute to virus replication in chickens. This possibility may at least partially explain why CL lipo treatment caused no significant decrease in virus load in the tissues. Second, NDV has tropism to various cell types, and macrophage reduction may lead to compensatory increases in virus replication in other cells, and thus the overall virus replication was not affected. Similarly, macrophage depletion resulted in more efficient infection of dendritic cells with measles virus, which increased virus load in the spleen in mice [30]. Third, macrophage depletion may impair the clearance of the virus-infected cells, promoting virus dissemination in chickens.

On the other hand, macrophage depletion markedly deteriorated tissue damage in the spleen caused by NDV. A genotype VII strain was used in this study, and it produces severe necrosis in the lymphoid tissues in chickens [16,31]. Many previous studies revealed that intense cytokine response contributes to pathology induced by genotype VII NDV infection [16,17,18]. Macrophages produce cytokines after virus infection. Thus, it seems to be plausible that macrophage depletion may lead to decreased production of cytokines and, thus, impairment of tissue injury. However, our findings showed that macrophage depletion resulted in marked aggravation of tissue damage caused by JS5/05. We speculated that virus infection and spreading in the spleen may accelerate because of the reduced amount of macrophages. In addition, NDV infection induces apoptosis, which is associated with tissue lesions. Macrophages function to engulf and clear virus-induced apoptotic cells. NDV infection after macrophage depletion enhanced splenic pathology, which may be also related to the impaired capability to clear apoptotic and necrotic cells induced by virus infection due to the decreased amount of macrophages. These findings were consistent with the results for many other pathogens, including measles virus, H3N2 influenza virus, MERS-CoV and MDV [27,28,29,30]. Therefore, macrophages function to protect against NDV-mediated pathology. CL lipo treatment causes macrophage depletion through inducing cell apoptosis, and previous studies demonstrated that NDV infection can also trigger apoptosis of macrophages [5,23]. Thus, it is reasonable to speculate that genotype VII NDV may induce apoptosis of macrophages, which undermines the host antiviral response and promotes tissue damage caused by the virus. Further studies are required to investigate the effect of NDV-induced cell death of macrophages on viral pathogenicity.

## 6. Conclusions

Our study demonstrated that genotype VII NDV had high tropism to macrophages, whereas the contribution of these cells to virus replication could be compensated for by other cells when they were depleted. Alternatively, macrophages played an important role in alleviating NDV-induced tissue damage. Macrophage depletion exacerbated virus-induced pathology without impairing virus replication. Our findings identified new roles of macrophages in NDV replication and pathogenicity in chickens.

## Figures and Tables

**Table 1:** Scoring of the histopathology changes of the spleen.

Group	dpi ^a^	Lesion Severity ^b^
PBS liposomes	1	+
	3	+++
Clodronate liposomes	1	++
	3	++++
Control	1	−
	3	−

^a^ dpi: day post infection; ^b^ scoring standards for tissue lesions: −, normal; +, mild to moderate lymphocyte proliferation; ++, moderate to marked lymphocyte depletion and necrosis; +++, marked lymphocyte depletion, necrosis and tissue hyperplasia; ++++, extensive and severe lymphocyte depletion and necrosis.

## Data Availability

Not applicable.
